# From a biomechanical perspective: pathogenesis, clinical manifestations and treatment strategies of adolescent idiopathic scoliosis

**DOI:** 10.3389/fped.2025.1649483

**Published:** 2025-10-16

**Authors:** Dongmei Li, Haokun Mo, Siying Yang

**Affiliations:** ^1^ Department of Pediatrics, West China Second University Hospital, Sichuan University, Chengdu, China; ^2^ Key Laboratory of Birth Defects and Related Diseases of Women and Children (Sichuan University), Ministry of Education, Chengdu, China; ^3^ Department of Orthopedics, Tongji Hospital, Tongji Medical College, Huazhong University of Science and Technology, Wuhan, Hubei, China

**Keywords:** adolescent idiopathic scoliosis, biomechanics, pathogenesis, clinical manifestations, treatment strategies

## Abstract

**Purpose:**

Adolescent Idiopathic Scoliosis (AIS) is a complex three-dimensional spinal deformity, and its etiology and progression mechanisms have not been fully elucidated. This review aims to comprehensively explain the pathogenesis, clinical manifestations, and treatment strategies of AIS from a biomechanical perspective, providing a new theoretical framework for clinical diagnosis and treatment.

**Methods:**

This review strictly follows the PRISMA guidelines for systematic literature search and selection. The search databases include PubMed, Embase, Web of Science, and Cochrane Library, with the cutoff date being June 2025. The search strategy involves a combination of keywords related to AIS, biomechanics, pathogenesis, clinical manifestations, and treatment. By progressively screening titles, abstracts, and full-text articles, relevant high-quality studies were ultimately included for comprehensive analysis.

**Results:**

The pathogenesis of AIS can be conceptualized as a “vicious cycle” driven by the interactional imbalance between passive subsystems (skeletal-ligament), active subsystems (muscles), and neurocontrolled subsystems (central and peripheral nerves). Biomechanical factors play a key role in driving the progression from initial minor imbalances to significant three-dimensional deformities. Clinically, symptoms such as body deformity, back pain, and reduced cardiopulmonary function can all be directly interpreted from a biomechanical perspective. In terms of treatment, all mainstream interventions (including observation, specific exercise rehabilitation, bracing, and surgery) fundamentally rely on biomechanical correction.

**Conclusion:**

The biomechanical perspective provides an indispensable integrative framework for understanding AIS. It unifies the process from molecular abnormalities to macro deformities, linking the diverse clinical manifestations and treatment approaches. Further exploration of biomechanical mechanisms is of significant importance for optimizing treatment timing and improving long-term patient outcomes.

## 1 Introduction

Scoliosis is a spinal deformity characterized by lateral curvature or vertebral rotation of one or more spinal segments. Idiopathic scoliosis, the most prevalent form, can manifest at any age. However, due to the rapid skeletal growth during puberty, it is most commonly diagnosed during adolescence. AIS has an unclear etiology, which poses significant challenges for clinical diagnosis and treatment. Traditional studies have focused on factors such as genetic predisposition, hormonal secretion abnormalities, and neurological control; however, the critical role of biomechanics in driving the occurrence and progression of AIS has not been systematically elucidated. In this review, we summarize and analyze the pathogenesis, clinical manifestations and treatment strategies of AIS from a biomechanical perspective. By examining AIS through a biomechanical lens, we can understand the fundamental mechanical principles underlying the condition and establish a theoretical basis for evaluating existing methodologies, developing novel intelligent braces, and creating mechanobiological intervention strategies. This article aims to provide clinicians with an integrated biomechanical framework to advance the standards of AIS diagnosis and treatment.

## 2 Materials and methods

This review strictly follows the guidelines of systematic reviews and PRISMA. Literature searches were conducted in the PubMed, Embase, Web of Science, and Cochrane Library databases, covering the period from the inception of the databases to December 2024. The search strategy combined both medical subject headings (MeSH) and free-text terms, including the following keywords: “adolescent idiopathic scoliosis,” “biomechanics,” “pathogenesis,” “clinical manifestations,” “treatment strategies,” and their combinations.

Study selection was carried out independently by two researchers. Duplicate articles were first excluded, and then titles and abstracts were screened according to predefined inclusion and exclusion criteria, followed by a full-text assessment of potentially eligible studies. Inclusion criteria included: (1) studies addressing the biomechanics of AIS; (2) studies involving pathogenesis, clinical manifestations, or treatment methods; (3) studies containing biomechanical parameters or analysis. Exclusion criteria were: (1) studies not focused on idiopathic scoliosis; (2) conference abstracts or case reports.

Data extraction was performed using a standardized form, which included the following information: first author, publication year, study type, sample characteristics, biomechanics assessment methods, main findings, and conclusions. Two reviewers independently extracted data, and any disagreements were resolved through discussion or consultation with a third researcher. Due to substantial heterogeneity among studies, a meta-analysis was not conducted, and a narrative synthesis method was employed to systematically summarize the results.

## 3 Results

### 3.1 Pathogenesis

#### 3.1.1 Fully upright walking

**Figure 1:**
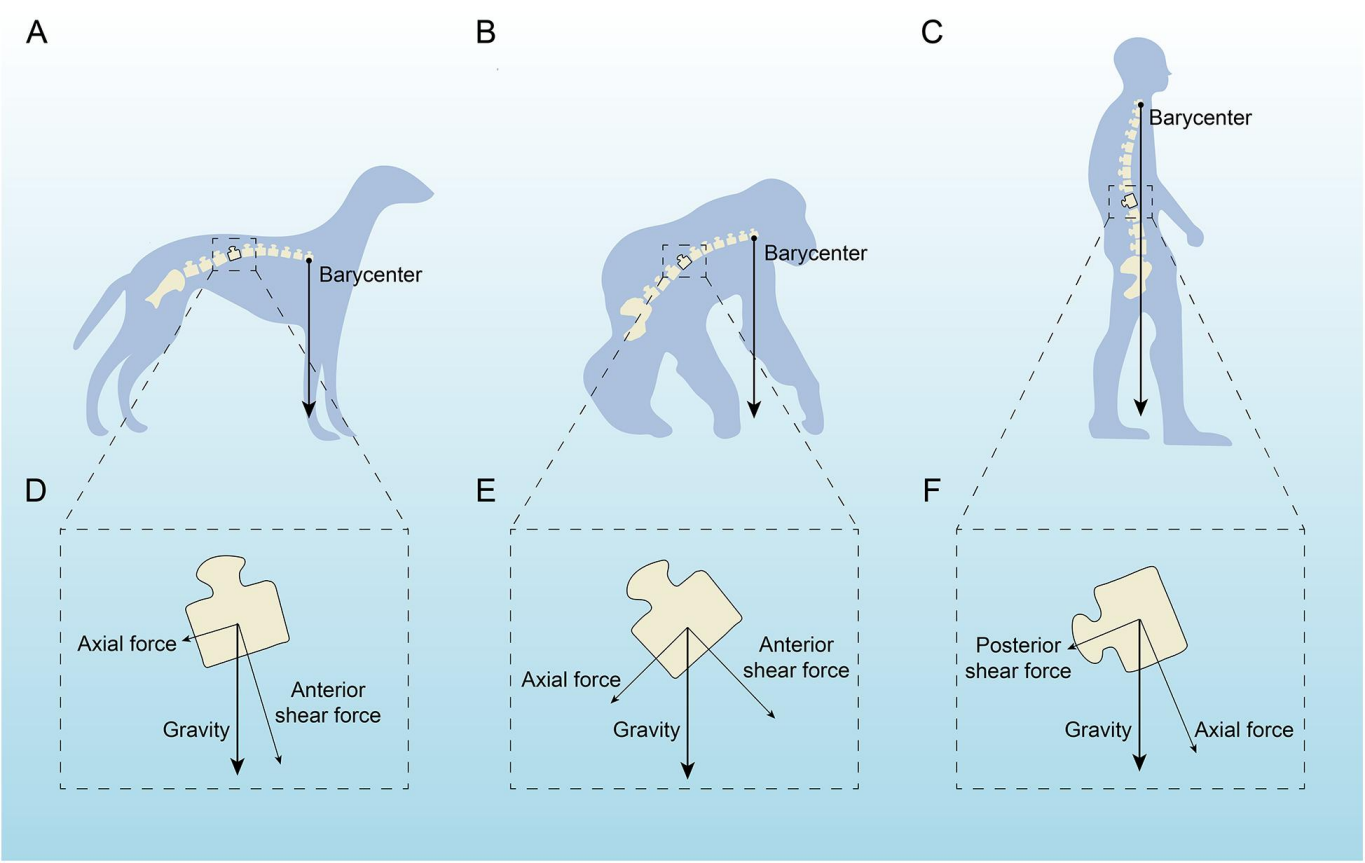
Analysis of the force acting on a single vertebra in dogs, apes and humans. **(A,D)** A quadruped with the barycenter near the hind legs, highlighting axial and anterior shear forces. **(C,F)** An intermediate stanced primate with similar force distribution but a slightly shifted barycenter. **(C,F)** An upright human, showing a central barycenter with axial and posterior shear forces affecting the spine. Arrows indicate force directions relative to gravity.

In terms of anatomical structure, the human spine is similar to the spines of many vertebrates, such as chimpanzees, cattle, rats, and mice (1–3). However, among all vertebrates, only humans develop AIS. Some studies suggest that bipedalism and the upright posture are closely related to AIS. Bipedalism and the upright posture lead to a series of changes in the human spine and trunk in three-dimensional space, affecting sagittal shape, axial pelvic-spinal rotation, and transverse plane reverse rotation (4, 5). Under normal physiological conditions, the arrangement of sagittal spinal tissues is strictly regulated, with children and adolescents undergoing changes in spinal alignment during growth and development while maintaining alignment between the spinal and lower limb joint centers (6). Under abnormal physiological conditions, abnormal spinal alignment and reduced rotational stability can lead to spinal deformities such as scoliosis (5, 7). Studies have shown that rats, which walk bipedally and must remain upright, develop scoliosis after pinealectomy, whereas quadrupedal rats with similar pinealectomy do not (8). We performed a force analysis on the spines of dogs (fully quadrupedal), apes (brief bipedal), and humans (fully bipedal), finding that the spines of quadrupedal animals and apes are more horizontally positioned, with the center of gravity located in front of the pelvis, while the human spine is closer to the vertical direction, with the center of gravity above the pelvis (Figure 1). Among all vertebrates, including humans, the spine primarily supports axial compression, which is mainly borne by the anterior column (intervertebral discs, endplates, and vertebral bodies) (9). Due to fully upright walking, the human spine bears a greater gravitational load, which affects the vertical growth of the spine. During growth and development in children and adolescents, incorrect upright and seated postures may form, leading to asymmetric distribution of gravitational load on the spinal epiphyseal plates, ultimately causing asymmetric vertebral growth and wedging, inducing scoliosis (10–12). In this review, we performed a force analysis on individual vertebrae of dogs, apes, and humans (Figure 1), finding that shear loads in the vertebrae of dogs and apes mainly direct toward the abdominal side, which can be countered by the pulling forces of small joints, muscles, and spinal ligaments, keeping the spine in a very stable state (7). In human vertebrae, some vertebrae experience shear loads directed toward the abdominal side, while others experience loads directed toward the back. The human spine is unable to effectively counteract the backward shear loads, leading to asymmetric loading, asymmetric growth, and axial rotational instability, which may result in the occurrence and development of scoliosis (7, 13–15).

#### 3.1.2 Regulatory factors

Panjabi proposed the concept of spinal stability through the “Three-Segment Model,” which delineates the spinal stability system into three components: a passive subsystem made up of ligaments and bones, an active subsystem comprised of spinal-associated muscles, and a neural control subsystem (16, 17). Abnormalities in any of these subsystems can lead to biomechanical alterations in the spine, thereby undermining its stability and potentially contributing to the development of AIS (Table 1). The proper growth, development, and functioning of bones, ligaments, muscles, and nerves within the spinal stability system depend on the expression of specific proteins. Disruptions in the expression of these proteins may result in the onset of AIS.

**Table 1:** Regulatory factors affect the biomechanical balance of the spinal stabilization system.

The spinal stability system	Regulatory molecules	Target cells/tissues	Biological effects	Biomechanical effects	References
Passive subsystem (bones)	PKM2↑	Human BM-MSCs	Osteogenic differentiation of bone marrow mesenchymal stem cells is weakened.	Decreased bone mass and weakened bone strength.	(31)
	Annexin A2↓	Human BM-MSCs, human osteoblasts	Osteogenic differentiation of bone marrow mesenchymal stem cells is weakened. The activity of ALP is reduced, and the mineralization of osteoblasts is weakened.	Decreased bone mass and weakened bone strength.	(31, 32)
	Phosphorylated HSP27↓	Human BM-MSCs, human osteoblasts	Osteogenic differentiation of bone marrow mesenchymal stem cells is weakened, and the mineralization of osteoblasts is weakened.	Decreased bone mass and weakened bone strength.	(33)
	Melatonin↓	Human BM-MSCs, human osteoblasts, human osteoclasts	Osteogenic and chondrogenic differentiation of bone marrow mesenchymal stem cells is weakened. Differentiation of osteoblasts is impaired and osteoclast differentiation is enhanced.	Decreased bone mass and weakened bone strength.	(37–41)
	Leptin↓	Human BM-MSCs	Osteogenic differentiation of bone marrow mesenchymal stem cells is weakened.	Decreased bone mass and weakened bone strength.	(43, 44)
	Estrogen ↓	Human osteoblasts, human osteoclasts	Promote osteoblast differentiation, inhibit osteoblast apoptosis, and promote osteoclast apoptosis.	Decreased bone mass and weakened bone strength.	(45, 46)
Active subsystem (muscles)	Tent5a is differentially expressed in paravertebral muscles.	Mouse myoblasts	The proliferation, migration and differentiation of paravertebral myoblasts were different, and the maturation of paravertebral type I myofibers was different.	The paravertebral muscles are asymmetrical, and the stress around the spine is unbalanced.	(50)
	PAX3 is differentially expressed in paravertebral muscles.	Muscle tissue	The proliferation, migration, and differentiation of paravertebral myocytes were different.	The paravertebral muscles are asymmetrical, and the stress around the spine is unbalanced.	(51)
	ADGRG6↓	Mouse tendon cells	The density of tendon fibers decreases, and the distribution of elastic fibers is uneven.	The elastic modulus of tendons and the breaking stress are reduced, and the stress around the spine is unbalanced.	(53)
	Calmodulin is differentially expressed in paravertebral muscles.	Human muscle cells	The regulation of myocytes by calcium ions is differentiated.	The contraction strength of the paravertebral muscles varies, and the stress around the spine is unbalanced.	(54)
	Estrogen receptors are differentially expressed in paravertebral muscles.	Human muscle cells	Paravertebral myocyte differentiation and apoptosis are different.	The paravertebral muscles are asymmetrical, and the stress around the spine is unbalanced.	(55, 56)
Neural control subsystem (nervous tissue)	UNCX↑	Zebrafish embryos	The primary motor neuron branches in the tail are abnormal, and the nerves in the trunk are defective.	The nervous system regulates an imbalance in the state of spinal alignment.	(59)
	SSPO↑	Zebrafish brain tissue	Affects the function of Reissner's fibers, cerebrospinal fluid homeostasis, neurogenesis and abnormalities in embryonic morphology.	The nervous system regulates an imbalance in the state of spinal alignment.	(60)

Abnormal skeletal growth and development are crucial mechanisms underlying AIS. The human spine normally maintains a specific mechanical balance to support essential life functions. Abnormalities in vertebral growth, such as accelerated or slowed bone development, excessive or insufficient bone volume, and variations in bone density, can disrupt the bone's mechanical properties, including strength, toughness, and stiffness, potentially leading to AIS. Compared to healthy individuals, patients with AIS exhibit accelerated skeletal growth (18–20) and reduced bone density (21, 22). Additionally, AIS patients experience asymmetrical load distribution at the vertebral epiphyseal plates, which causes uneven vertebral growth and wedging (23, 24). Research indicates that in AIS patients, there is a disturbance in the growth balance between the anterior column (including intervertebral discs, endplates, and vertebrae) and the posterior column (including the vertebral arch and facet joints), with a tendency for overgrowth in the anterior column, resulting in asymmetric spinal development (25–28). Recent studies have shown that not only the abnormal development of vertebral bodies can lead to the occurrence of AIS, but also the deformities of the ribs and thoracic cage ultimately contribute to AIS (29). Abnormal growth and development of the ribs and thoracic cage will subsequently affect the intervertebral discs, and ultimately, the vertebral bodies will deform due to asymmetric loads, muscle forces, growth, and gravity. The growth and development of spinal bones are regulated by various growth-related proteins, which influence bone development and, consequently, the mechanical properties such as strength, toughness, and stiffness. The process begins with bone marrow mesenchymal stem cells, which have high proliferative potential and can differentiate into osteoblasts, chondrocytes, or adipocytes (30, 31). In proteomic studies of bone marrow mesenchymal stem cells (BM-MSCs) from AIS patients, a significant upregulation of pyruvate kinase M2 (PKM2) has been observed. PKM2 negatively correlates with cell differentiation, resulting in weakened osteogenic differentiation of BM-MSCs, inhibited skeletal growth and development, decreased bone mass, and reduced bone strength (32). Additionally, annexin A2 is found to be significantly downregulated in mesenchymal stem cells (MSCs) from AIS patients. Annexin A2 promotes osteogenic differentiation of BM-MSCs, enhances alkaline phosphatase (ALP) activity, and supports mineralization of osteoblasts. Its downregulation is associated with lower bone density and compromised bone strength (32, 33). Moreover, a marked reduction in phosphorylated heat shock protein 27 (HSP27) levels has been reported in osteoblasts and BM-MSCs of AIS patients. Upregulation of phosphorylated HSP27 in these cells promotes the expression of bone formation markers (34). Melatonin deficiency has been shown to induce scoliosis in pinealectomized chickens and mice (35, 36). AIS patients also exhibit dysfunction in melatonin signaling (37). Melatonin, primarily produced by the pineal gland, enhances osteogenic and chondrogenic differentiation of BM-MSCs (38, 39), stimulates osteoblast differentiation, and promotes osteogenesis (40). Furthermore, melatonin inhibits the binding of osteoclast differentiation factors to the receptor activator of nuclear factor-*κ*B (RANK), reducing osteoclast differentiation (41) and thereby increasing bone density and strength (42). In AIS patients, a downregulation of leptin receptors in BM-MSCs has been observed, potentially leading to decreased sensitivity to circulating leptin (43). Leptin directs BM-MSCs towards osteogenic pathways rather than adipogenic ones, enhancing bone density and strength (44, 45). Estrogen also impacts the development of scoliosis. Its primary role in skeletal development is mediated through estrogen receptor alpha. Estrogen's most significant effects on bone metabolism likely involve promoting osteoblast differentiation, inhibiting osteoblast apoptosis, and facilitating osteoclast apoptosis, thus enhancing skeletal growth and bone strength (46, 47).

Research into the paravertebral muscles reveals that changes and asymmetries in these muscles impair spinal posture and movement control, contributing to the progression of AIS. Studies have identified variations in the biomechanical properties of these muscles in AIS, including differences in muscle tension, stiffness, relaxation time, and the Deborah number (indicative of creep). These biomechanical characteristics are linked to the severity of scoliosis (48, 49). The alterations and asymmetries in the paravertebral muscles are associated with the expression of specific genes. Tent5a, an atypical poly(A) polymerase, has been identified as a susceptibility gene for AIS (50). It shows differential expression in the bilateral paravertebral muscles of AIS patients. Inhibiting Tent5a expression can reduce myoblast proliferation, migration, and differentiation, and also inhibit the maturation of type I muscle fibers, leading to decreased muscle asymmetry, improved spinal stress balance, and reduced scoliosis progression (51). Furthermore, PAX3 expression in the paravertebral muscles of AIS patients varies between sides, with a positive correlation between PAX3 levels and muscle volume. The convex side exhibits higher PAX3 expression and muscle volume compared to the concave side, causing an imbalance in the forces exerted on the spine (52). ADGRG6, a member of the adhesion G protein-coupled receptor family, shows a strong association with AIS (53). Inhibition of ADGRG6 leads to decreased tendon fiber density and uneven elastic fiber distribution, lowering elastic modulus and failure stress, thus disrupting spinal stress balance (54). Calmodulin, a crucial calcium-binding protein, regulates calcium signaling and skeletal muscle contraction. In AIS patients, calmodulin is unevenly distributed in the paravertebral muscles, with higher levels on the convex side and lower on the concave side (55). This disparity in contraction strength between the bilateral paravertebral muscles disrupts spinal stress balance. Estrogen influences skeletal muscle activity and regulates vascular endothelial growth factor production, which supports muscle cell differentiation and anti-apoptotic processes (56). In female AIS patients, estrogen receptor type 1 (ESR1) and type 2 (ESR2) show asymmetrical expression in deep paravertebral muscles, with the extent of this asymmetry positively correlated with Cobb angle and progression risk factors (57).

Abnormalities in the nervous system are crucial to the pathogenesis of AIS. The pathogenesis of AIS primarily involves the extrapyramidal system. Numerous studies have demonstrated that dysfunction in afferent nerves, the regulatory center, and efferent nerves can all contribute to the development of AIS (58–60). Regarding afferent pathways, research has shown that AIS patients experience a significant decline in balance while standing with their eyes closed. Additionally, their somatosensory evoked potentials exhibit abnormal latencies and amplitudes, suggesting impaired signal processing in the spinal cord's dorsal columns or the somatosensory cortex (58). Additionally, some AIS patients present with cerebellar tonsil ectopia, which is associated with more severe spinal curvature (61). The biomechanical impact of gene expression in human neural tissue on AIS remains unexplored. However, studies in zebrafish have demonstrated that modulating the expression of certain genes within neural tissue can influence AIS development. For example, overexpression of UNCX in zebrafish larvae results in abnormal branching of primary motor neurons in the tail, which leads to defects in trunk nerve development and disrupts the spinal alignment normally regulated by the nervous system, causing spinal curvature (62). Moreover, irregular deposition of zebrafish scospondin (sspo) within the brain ventricles has been linked to idiopathic-like scoliosis. sspo influences the function of Reissner's fiber, which regulates cerebrospinal fluid homeostasis, neurogenesis, and embryonic development. Inhibiting the irregular deposition of sspo helps maintain spinal balance and alignment (63). In terms of efferent pathways, dysfunction is primarily observed as an imbalance in the excitability of spinal α-motor neurons and associated neuromuscular control deficits. Electromyography studies have revealed that AIS patients' paraspinal muscles display asymmetric electrical activity both at rest and during movement, with abnormal muscle excitability patterns on the convex and concave sides (60). This abnormality may stem from changes in the recruitment sequence and rate of motor neuron pools, leading to an imbalance in muscle strength required to maintain spinal symmetry. During the growth period, this imbalance applies asymmetric stress to the spine, which exacerbates its three-dimensional deformity.

AIS is likely not initiated by a single subunit, but rather a minor defect in one subunit that triggers a positive feedback loop. A slight abnormality in neural control (such as a minor proprioceptive defect) causes the patient to unconsciously adopt a slightly asymmetric posture when standing or moving. Alternatively, a subtle ligamentous laxity in the passive subunit allows the spine to undergo slight deformation more easily under external forces. This initial asymmetric posture leads to biomechanical changes in the spine. In order to maintain this unstable curved posture, the muscles are compelled to compensate. The concave side muscles are forced to contract continuously, resulting in fatigue, spasms, and functional degradation. The convex side muscles are overstretched, further exacerbating the imbalance in muscle strength and coordination. The deformed spine and abnormally functioning muscles send erroneous feedback signals to the neural control system, prompting it to issue increasingly inaccurate commands for muscle control, thereby reinforcing and worsening the faulty posture, eventually leading to progressive, structural three-dimensional spinal deformity. Panjabi's “Three-Segment Model” provides an excellent biomechanical integrative framework for understanding AIS. It reveals that AIS is not a static deformity but rather a dynamically progressing process driven by the imbalance of interactions within the neuromuscular-skeletal system. This calls for a clinical practice that transcends mere orthotics, advocating for a comprehensive treatment strategy aimed at restoring the functionality of the entire spinal system.

### 3.2 Clinical feature

#### 3.2.1 Somatic deformity

**Figure 2:**
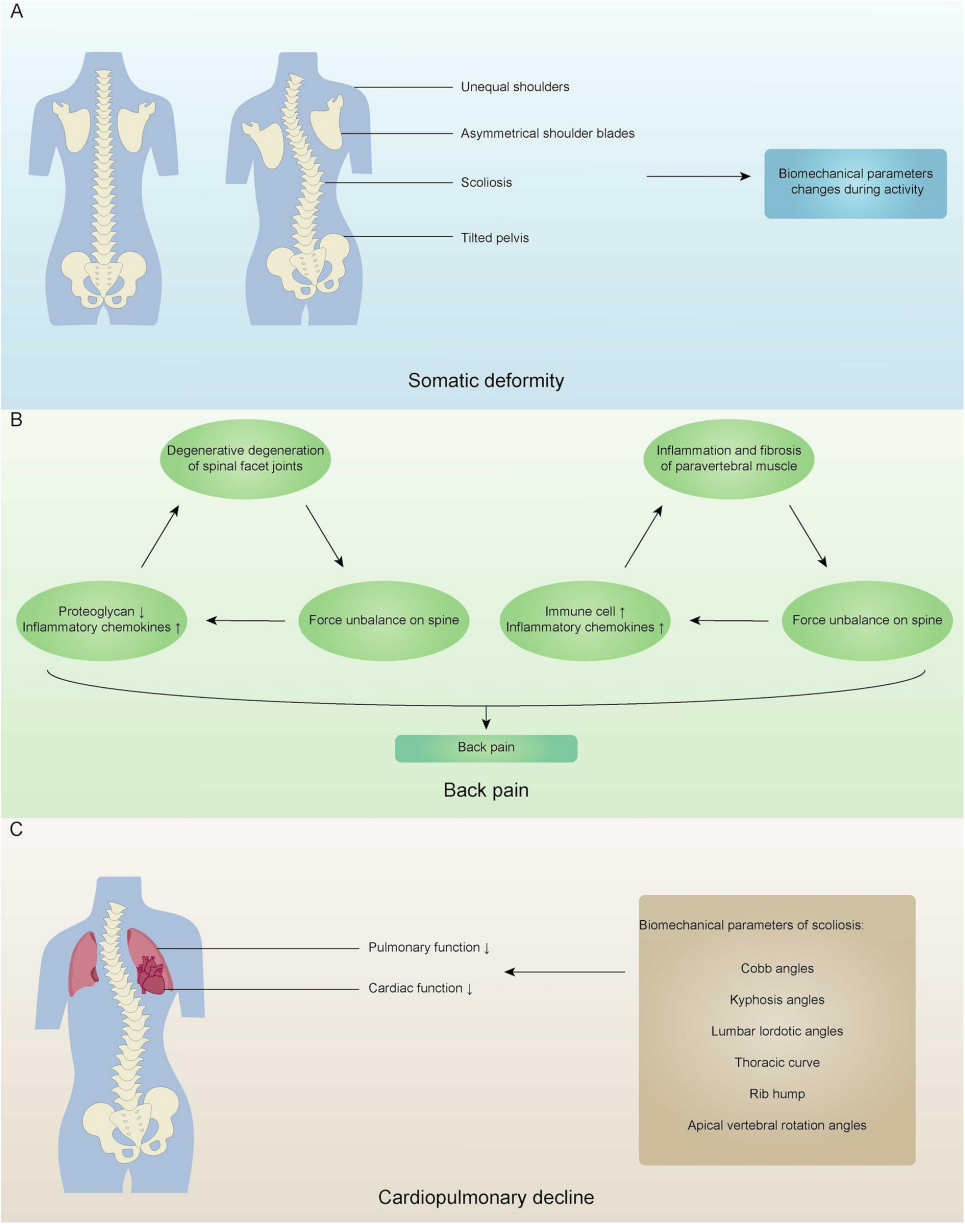
Clinical manifestations of adolescent idiopathic scoliosis. **(A)** A normal spine and a spine with somatic deformity, highlighting unequal shoulders, asymmetrical shoulder blades, scoliosis, and tilted pelvis, leading to changes in biomechanical parameters during activity. **(B)** The cycle resulting in back pain, involving degeneration of spinal facet joints, force imbalance on the spine, inflammation of paravertebral muscle, and changes in proteoglycans and inflammatory chemokines. **(C)** Cardiopulmonary decline due to decreased pulmonary and cardiac function, with biomechanical parameters of scoliosis such as Cobb angles, kyphosis angles, and others.

AIS primarily involves one or more vertebral segments bending laterally with associated vertebral rotation, typically developing in adolescents during or around puberty. Mild scoliosis often does not cause significant discomfort or noticeable somatic deformity (64). In contrast, more severe scoliosis can impact adolescent growth and development, leading to noticeable physical deformities and structural abnormalities (65) such as uneven shoulders, asymmetrical scapulae, spinal deviation from the midline, and pelvic tilt (Figure 2A). From a static biomechanical perspective, the rotating vertebrae induce structural deformation in the attached ribs, leading to the clinically visible “scapular prominence” deformity and thoracic asymmetry (66). This disrupts the alignment between the two mechanical bases: the thoracic cage and the pelvis. In order to maintain a centered head position and horizontal eye alignment as much as possible in the sagittal and coronal planes, the body compensates by modifying spinal curvature and pelvic tilt (67). However, this compensatory mechanism is often imperfect, resulting in external signs such as uneven shoulders and scapular asymmetry. Additionally, the normal physiological curvature of the spine is frequently disturbed, further reducing the spine's efficiency in absorbing and dispersing stress, thereby making it more vulnerable to axial loading. These structural changes result in altered biomechanical performance in patients with AIS compared to healthy individuals. Research indicates that AIS patients exhibit reduced hip and pelvic movement during walking, excessive energy expenditure, asymmetric gait patterns, and uneven ground reaction forces (68). During physical activity, a deformed spine, as a dysfunctional movement chain, exhibits significant reductions in range of motion, flexibility, and energy transmission efficiency. Research has confirmed that patients with AIS show distinct biomechanical patterns during walking: the kinematic parameters of the hip joint and pelvis in the horizontal and coronal planes are reduced, which is a compensatory strategy aimed at reducing spinal movement and maintaining trunk stability (69). However, this strategy comes at the cost of reduced movement economy, leading to increased energy expenditure and greater susceptibility to fatigue (69). Additionally, due to changes in the biomechanical advantages of the trunk muscles (such as abnormal length-tension relationships between the convex and concave side muscles) and disturbances in proprioceptive input, patients exhibit step asymmetry and uneven ground reaction force distribution throughout the gait cycle (70).

#### 3.2.2 Back pain

Back pain is a significant clinical feature of AIS. Compared to the control group, patients with AIS experience a higher prevalence of back pain, which is not only more severe but also lasts longer and recurs more frequently (64, 71–73). The etiology of back pain in these patients is multifactorial, involving both biochemical and biomechanical mechanisms (74). Research indicates that AIS patients exhibit notable degenerative changes in the spinal facet joints, including considerable loss of proteoglycans, increased cell density, elevated levels of pro-inflammatory markers, and the presence of prominent small leucine-rich proteoglycan (SLRP) fragments, such as chondroadherin and decorin (75). Facet joints are crucial for maintaining spinal stability, and their degenerative changes are widely recognized as a source of pain in chronic back pain cases (76, 77). When facet joints undergo degeneration, the spine loses its force balance and develops rotational instability, which exacerbates the degeneration of the facet joints and results in significant back pain in AIS patients (78). In a separate prospective study, Samaan et al. found that pain is associated with inflammation of the paravertebral muscles (79). In AIS, muscle inflammation is a process that involves both acute and chronic stages. The repeated cycles of muscle tissue damage associated with the progression of scoliosis may lead to inflammation and fibrosis. Furthermore, paraspinal muscles undergo structural remodeling in response to abnormal mechanical environments. The muscles on the concave side of the spine demonstrate more pronounced fibrosis and fat degeneration, while those on the convex side experience micro-damage and inflammatory responses due to compensatory overuse (80). Consequently, the force exerted by the paravertebral muscles becomes unbalanced, leading to rotational instability in the spine and further increasing inflammatory chemokines and immune cells in the muscle tissue, thus creating a vicious cycle and resulting in significant back pain (79).

#### 3.2.3 Cardiopulmonary decline

As AIS progresses, it adversely affects both pulmonary and cardiac functions. Numerous studies have documented a decline in pulmonary function in patients with AIS (81–83). This decline is often attributed to factors such as distortion of the spine and thoracic cavity, spinal compression on the lungs, secondary rib deformities, and decreased mobility of the chest wall (84–86). From a biomechanical standpoint, the primary cause of reduced pulmonary function in patients with AIS is the compression and restriction imposed by the deformed spine and thoracic cavity. The combination of vertebral rotation and rib hump associated with scoliosis leads to asymmetric collapse of the thoracic cage, significantly reducing the thoracic volume, particularly on the convex side. This structural change impairs pulmonary function through two main mechanisms. Firstly, restrictive ventilatory dysfunction, which results from a decrease in chest wall compliance and limited diaphragm movement, jointly hampers lung expansion. Secondly, local compression of lung tissue can lead to a mismatch between ventilation and perfusion, affecting the efficiency of gas exchange. Researchers have extensively investigated the relationship between spinal deformity parameters and pulmonary function in AIS patients. Recent studies have found that the pulmonary function of patients with AIS is negatively correlated with the Cobb angle. A significant inverse relationship exists between the Cobb angle and both the absolute and predicted values of forced expiratory volume in 1 s, forced vital capacity, vital capacity, and total lung capacity. The results indicate that for every 2.6–4.5 degrees of spinal curvature, a 1% decline in predicted lung function is observed (87). However, because AIS is a three-dimensional spinal deformity, evaluating pulmonary function based solely on the Cobb angle is insufficient. Research has shown that worse pulmonary function is associated with larger proximal and main thoracic Cobb angles, decreased kyphosis angles, increased lumbar lordosis angles, longer thoracic curvature, larger rib humps, greater apical vertebral rotation, and a smaller thoracic cage (88). As AIS advances, severe impairment of pulmonary function can lead to reduced lung volumes, resulting in pulmonary arterial hypertension and impaired cardiac function (89). Furthermore, a correlation has been observed between right ventricular function and the Cobb angle, with severe cases of AIS showing limited right ventricular systolic function (90).

### 3.3 Therapy

**Figure 3:**
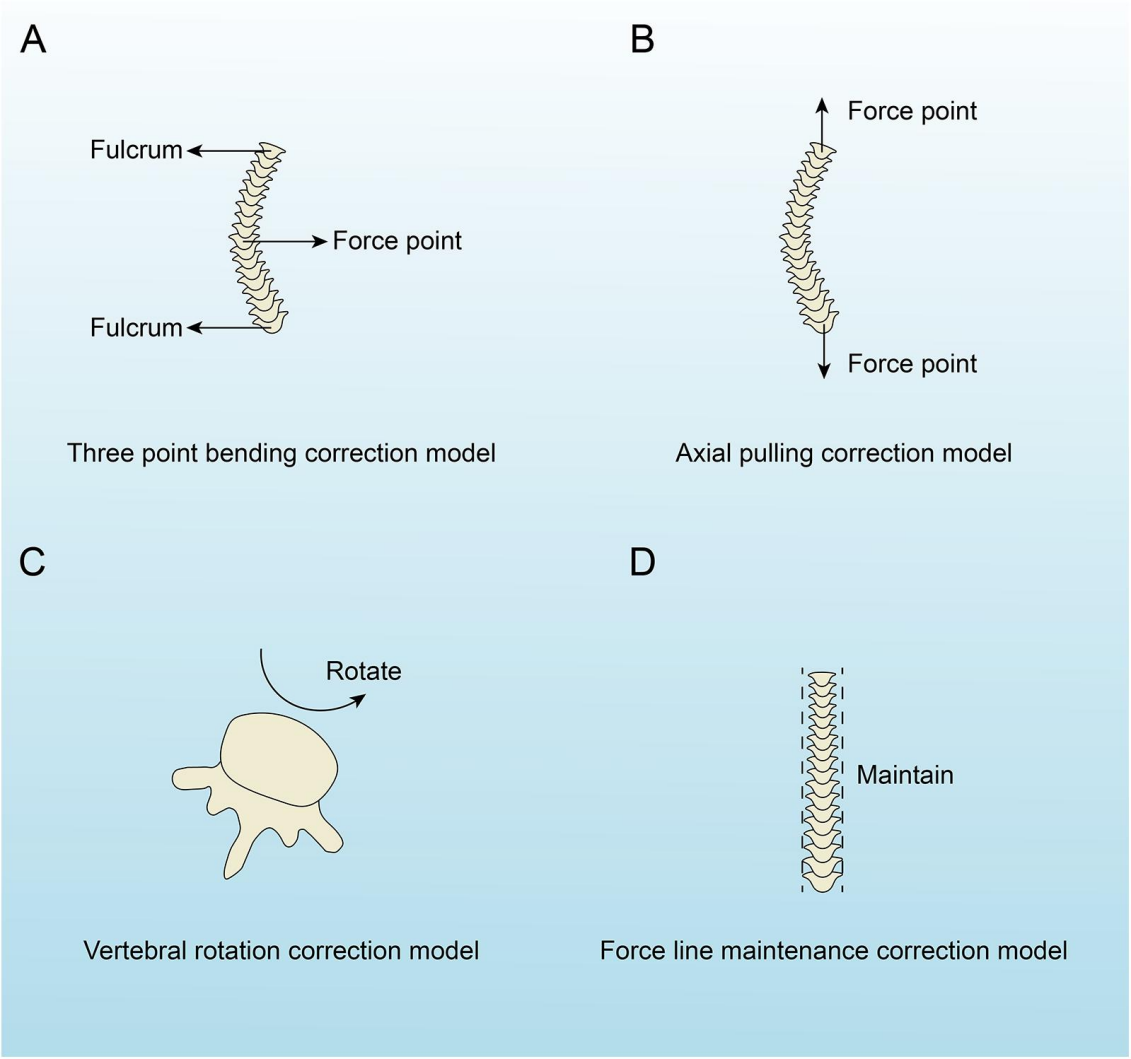
The four biomechanical correction models of adolescent idiopathic scoliosis. **(A)** Three-point bending model with labeled fulcrum and force point. **(B)** Axial pulling model with two force points. **(C)** Vertebral rotation model indicating rotation. **(D)** Force line maintenance model with vertical alignment labeled “Maintain”.

The treatment methods for AIS primarily consist of non-surgical and surgical approaches. Non-surgical methods include exercise-based orthotic treatment and brace orthotic treatment, whereas surgical approaches involve traditional spinal fusion using rods and screws and growing rod surgery. We performed a biomechanical analysis of these treatment methods, categorizing them into four biomechanical correction models: the three-point bending correction model, the axial pulling correction model, the vertebral rotation correction model, and the force line maintenance correction model (Figure 3). The corrective treatment approaches for AIS incorporate one or more of these biomechanical correction models (Table 2).

**Table 2:** The corrective treatment approaches for adolescent idiopathic scoliosis.

Classification of therapy	Therapy	Mechanism				Study type	Sample size	Key outcomes	Merit	Shortcoming	References
		The three-point bending correction	The axial pulling correction	The vertebral rotation correction	The force line maintenance correction						
Exercise-based treatment	General exercise therapy (pull-ups, swimming, and yoga)	Yes	Yes	Yes	No	Systematic review	/	Rate of progression, Cobb angle	No surgery is required and there is little pain for the patient.	It is only suitable for mild scoliosis and has limited therapeutic effect.	(92)
	Schroth	Yes	Yes	Yes	No	Randomized controlled trial	50	Maximum curve			(95)
	Barcelona Scoliosis Physical Therapy School (BSPTS),	Yes	Yes	Yes	No	Systematic review	/	Cobb angle, pulmonary function			(101)
	Dobomed	Yes	Yes	Yes	No	Retrospective study	152	Cobb angle, apical vertebral rotation degree			(105)
	Side Shift	Yes	Yes	Yes	No	Retrospective study	69	Curve size			(108)
Bracing	The Milwaukee brace	No	Yes	No	Yes	Systematic review	/	Cobb angle, pulmonary function, apical vertebral rotation degree	No surgery is required, and good treatment results can be achieved.	It requires prolonged wear to achieve therapeutic effect, poor patient compliance, and poor treatment effect for severe scoliosis.	(119)
	The Boston brace	Yes	No	No	Yes	Retrospective study	50	Cobb angle, the amount of initial correction			(120)
	The Charleston brace	Yes	No	No	Yes	Retrospective study	95	Cobb angle, apical vertebra position, skeletal maturity			(121)
	The SpineCor brace	No	No	Yes	Yes	Retrospective study	195	Cobb angle			(123)
	The Sforzesco brace	No	No	No	Yes	Prospective cohort study	50	Cobb angle			(125)
Surgical intervention	Posterior spinal fusion	Yes	No	Yes	Yes	Systematic review	1,819	Usage rate of screw structure, blood loss volume, usage rate of antifibrinolytic agents, operation time	It is effective in the treatment of severe scoliosis and is the standard surgery for scoliosis surgery.	The growth and movement of confluent spinal segments are limited, and degenerative changes and arthritis may occur.	(127)
	Anterior vertebral body tethering	Yes	No	No	Yes	Systematic review	/	Preservation of range of motion, Cobb angle correction, quality of life, complications, as well as muscle strength and endurance	It is able to maintain the growth and flexibility of the spine while treating scoliosis.	It is only suitable for skeletal immature patients with mild curvature of the spine and has a higher surgical risk.	(151)
	Growing rod technique	No	Yes	No	Yes	Retrospective study	8	Cobb angle, spinal growth	It is able to complete the orthopedic spine while allowing the spine to continue to grow.	Conventional growth rod technique requires multiple surgeries, and magnetic growth rod technique limits the effectiveness of the treatment due to the decrease in magnetism.	(164)
	The SHILLA growth guidance system	Yes	No	Yes	Yes	Systematic review	21	Changes in curve apex, development of adjacent compensatory curves, vertical spinal growth	It is able to complete the orthopedic spine while allowing the spine to continue to grow.	Wear and tear of the screws and rods can create metal chips, which can lead to the creation of an inflammatory response.	(170)

/ indicates not applicable.

#### 3.3.1 Exercise-based treatment

Exercise-based treatments encompass general exercise and physiotherapeutic scoliosis-specific exercises (PSSE). General exercise therapy involves low-intensity stretching and strength training activities such as pull-ups, swimming, and yoga, which can help stabilize the spine and alleviate symptoms (91, 92). It is widely accepted that PSSE offers superior therapeutic benefits for AIS compared to general exercise. PSSE is a tailored exercise program designed according to the patient's spinal curvature, degree, and clinical characteristics, with an emphasis on preventing curvature progression (93). Various PSSE methods exist, all based on similar biomechanical principles. Here, we focus on four commonly utilized techniques: Schroth, Barcelona Scoliosis Physical Therapy School (BSPTS), Dobomed, and Side Shift. The method of Schroth involves specific postural correction, correction of breathing patterns, and correction of postural perception. This method strengthens the paravertebral muscles through targeted exercises, aiming for asymmetric sagittal plane alignment, axial elongation, and rotational breathing. These techniques are designed to halt curve progression, correct abnormal spinal curves, reduce pain, increase lung capacity, and improve overall posture and appearance (94, 95). The physical therapy strategies for the thoracic spine (particularly in the T2 to T10 segments) make full use of the biomechanical coupling effect between the ribs and thoracic vertebrae. The “rotational breathing” technique in the Schroth method consciously guides the expansion of the rib space on the convex side, which applies a persistent, lateral de-rotational torque to the connected vertebrae. This mechanically induced force transmitted through the ribs helps correct vertebral rotational deformities and, to some extent, improves the symmetry and mobility of the thoracic cage (96). Schroth, as one of the most representative forms of PSSE, has been strongly supported by extensive evidence from clinical research. In recent years, several high-quality systematic reviews and meta-analyses have consistently demonstrated significant benefits of this therapy in improving Cobb angle, trunk rotation, and quality of life. A meta-analysis by Ceballos-Laita et al. clearly indicated that, compared to conventional treatments, Schroth therapy significantly reduces the Cobb angle in patients with AIS (96). Further analysis by Baumann et al., through a systematic review and meta-analysis of randomized controlled trials, strengthened this conclusion by incorporating observational studies for subgroup analysis, confirming the positive role of Schroth therapy in controlling angle progression (97). In addition to structural parameters, Schroth therapy has also shown positive effects on improving patients' functional outcomes and health-related quality of life. The study by Ceballos-Laita et al. additionally reported significant advantages of this therapy in reducing trunk rotation angles and improving scores on quality of life questionnaires (96). A network meta-analysis by Wang et al. further confirmed these findings, showing long-term benefits of Schroth therapy in improving spinal deformities and quality of life among six specific exercise interventions compared (98). This suggests that its effectiveness extends beyond radiological indicators to include aesthetic improvements and psychosocial well-being, embodying the modern concept of comprehensive treatment. It is noteworthy that the effectiveness of Schroth therapy has been contextualized within the broader framework of exercise interventions. A network meta-analysis by Jiang et al. compared and ranked various PSSEs. Although all exercise regimens demonstrated some degree of effectiveness, the combination of Schroth therapy with core stabilization training was considered the optimal strategy for addressing AIS (99). Chen et al.'s study suggested that exercise interventions can significantly improve AIS, but the differences in improvement between different exercise forms (core strength training, PSSE, yoga, Schroth, and sling) were not statistically significant (100). Current evidence from clinical research consistently supports Schroth therapy as an effective, non-invasive treatment for improving the Cobb angle, trunk rotation, and quality of life in patients with AIS. BSPTS, an evolution of the method of Schroth, uses translation, rotation, and axial elongation exercises to prevent curve progression, correct scoliosis posture, stabilize the spine, and enhance respiratory function (101). BSPTS is typically applicable to patients with mild to moderate scoliosis or as an adjunct to brace treatment to enhance muscle strength and improve posture symmetry. Currently, high-level evidence from evidence-based medicine specifically targeting BSPTS remains relatively limited. Seleviciene et al. explored various physical therapy methods and their efficacy currently used for conservative treatment of AIS in a literature review. The review indicated that BSPTS, as one of the main approaches within PSSE, is clinically applied based on its systematic theoretical framework and clinical observations. However, the review also highlighted that despite the widespread use of PSSE (including BSPTS), research in this field still faces challenges, and many treatments require more high-quality studies to provide strong evidence for their efficacy (102). Future research should focus on identifying the optimal indications for BSPTS, treatment dosages (frequency and duration), and its unique advantages or specific therapeutic effects, thus providing guidance for its precise application in clinical practice. The method of Dobomed, also referred to as the Dobosiewicz's method, is a 3D biomechanical correction technique based on AIS pathomechanics, aiming to correct spinal curvature through 3D vertebral displacement (101). Evidence shows that the method of Dobomed can reduce Cobb angles and vertebral rotation while preventing further curvature progression (103–105). Currently, high-quality randomized controlled trials and traditional meta-analyses specifically targeting Dobomed therapy are relatively limited, which somewhat restricts the independent and precise evaluation of its efficacy. However, the study by Wang et al. systematically compared the efficacy of six different scoliosis-specific exercises for AIS (98). Since no available Dobomed clinical trials met the inclusion criteria, the efficacy of Dobomed therapy for AIS was not compared. Although the study may indicate that certain methods (such as Schroth) show relative advantages in statistical rankings, Dobomed therapy, as part of a comprehensive physical therapy system, still holds clinical value in improving posture and function in AIS patients, particularly for those with specific types of scoliosis, such as reduced thoracic kyphosis. Side Shift therapy includes active 3D automatic correction (in the transverse, coronal, and sagittal planes), excessive corrective movements beyond the midline, trunk shifting opposite the main curve, and repeated correction exercises during growth to influence spinal development (101, 106). Studies suggest that the Side Shift therapy effectively halts curvature progression and is a viable treatment for AIS (107, 108). Currently, evidence from evidence-based medicine specifically targeting the side-shifting method remains limited. A network meta-analysis conducted by Wang et al. compared the efficacy of six different scoliosis-specific exercises on AIS (98), but we cannot directly obtain quantitative comparison data or ranking results regarding the efficacy of the side-shifting method from this study. This situation itself indicates that the side-shifting method is usually incorporated into broader physical therapy programs for research, rather than being assessed as an independent intervention. Furthermore, core stability training may help suppress the progression of scoliosis by optimizing the dynamic stability of the spine and reducing asymmetrical loads applied to the spine during daily activities. Studies show that a scientifically designed core stability training program can be an effective component of PSSE, providing intrinsic biomechanical support for the spine (101). Liu et al. confirmed that core stability training could benefit mild to moderate AIS, but due to heterogeneity, small sample size, and multiple comparisons, the evidence for the efficacy of core stability training is limited (109). The network meta-analyses by Chen et al. and Jiang et al. both included core muscle training as an important control intervention when comparing various exercise interventions. Although these analyses may indicate that some more comprehensive PSSE methods (such as Schroth) are statistically superior, core training, as a foundational intervention, is often integrated into more comprehensive rehabilitation programs to enhance treatment effects (99, 100). The core of combined exercise programs is to maximize treatment effects through synergy. These programs may integrate various elements such as scoliosis-specific exercises (e.g., Schroth), core stability training, symmetry-strengthening exercises, and posture education, aiming to address the three-dimensional deformity and functional impacts of AIS from multiple dimensions. Currently, randomized controlled trials focusing on “combined exercise programs” as an independent intervention are relatively limited. A network meta-analysis by Li et al. compared the short-term effects of different strategies in conservative treatment for AIS, and the results reinforced the short-term efficacy of bracing combined with scoliosis-specific exercises. This study provides indirect but important support for the concept of “combined treatment,” suggesting that combining treatment methods with different mechanisms may be a more promising strategy to address the complexity of AIS through multi-target effects, thereby achieving better overall outcomes (110). In addition to its traditional roles in muscle strengthening and posture correction, physical therapy can also modulate the extrapyramidal system. Targeted proprioceptive training and balance exercises can enhance the functional activity of this system. Through repetitive and patterned motor training, the regulation of spinal motor neurons by structures such as the basal ganglia, cerebellum, and brainstem can be optimized. This process improves the balance and coordination of the paravertebral muscles, achieving greater precision and automaticity without the need for conscious control (111).

#### 3.3.2 Bracing

In the 16th century, the French surgeon Ambroise Pare was the first to employ metal orthotic devices for spinal deformities. Following this, numerous braces and their derivatives, such as the Milwaukee brace and the Lyon brace, were developed for scoliosis treatment (112–114). Brace orthopedic treatment is indicated for AIS with a Cobb angle ranging from 20°–40° (115, 116). Currently, there is a wide variety of braces used for AIS treatment. Negrini et al. proposed a classification system for spinal braces based on criteria including building, rigidity, anatomical classification, aonstruction of the envelope, mechanism of action, and plane of action (117). Although many braces share similar biomechanical mechanisms, we will focus on a few representative examples. The Milwaukee brace, the first widely used brace for scoliosis treatment globally, consists of a rigid orthotic frame designed to correct the curved spine through axial extension. However, due to the substantial discomfort it causes, patient compliance tends to be lower, making it advisable to use the Milwaukee brace only intermittently (118, 119). In 1972, the Boston brace was introduced, primarily constructed from metal, leather, and canvas. This brace features a symmetrical design with a posterior opening and is characterized as a rigid orthotic. Its biomechanical function is based on a three-point bending system, which has been shown to produce favorable therapeutic outcomes (115, 119). A controlled matched study evaluating the effectiveness of the Boston brace for managing greater curves in 50 skeletally immature female patients found that the Boston brace was effective in preventing progression of greater curves when used ≥18 h per day (120). The Charleston brace is a nocturnal scoliosis corrective brace made from a plaster mold, employing a three-point bending mechanism to correct spinal curvature. Studies indicate that the Charleston brace is highly effective in managing AIS, and its nocturnal use has been shown to improve patient compliance and reduce psychological stress to some extent (121). Asymmetric nocturnal braces (such as the Providence brace) facilitate the posterior migration of the nucleus pulposus towards the midline in the apex of the scoliosis curve. The brace exerts a continuous, localized lateral pressure on the convex side of the apex through precisely molded pressure pads, while providing corresponding release space on the concave side. This pressure induces gradual displacement of the nucleus pulposus under sustained low load, progressively correcting the asymmetric deformation of the intervertebral disc (122). The SpineCor brace is a flexible spinal orthosis that includes a rotational strap connecting the pelvis to the shoulder straps. This brace allows for dynamic adjustment of the rotational strap's tension, which is tailored according to the specific curvature patterns of the spine. Research suggests that the SpineCor brace can partially correct scoliosis (123). The Sforzesco brace is a custom-made thoracolumbar orthosis designed according to the SPoRT concept (Symmetric, Patient-oriented, Rigid, Three-dimensional, and Active). It utilizes thrust-based correction techniques for scoliosis and has been shown to have favorable treatment outcomes (124, 125). In the thoracic segments from T2 to T10, the pressure applied by the brace to the ribs is effectively transmitted to the corresponding vertebral bodies via the costovertebral and costotransverse joints, generating a powerful moment for de-rotation and lateral translation, which allows for three-dimensional correction of the thoracic deformity. The brace design often includes a frontal plane flexion mechanism with an opening on the anterior axillary line of the concave side. This design not only prevents excessive pressure on the concave side ribs but, more importantly, increases the volume of the concave thoracic cavity, thus creating physical space for the expansion of restricted lung tissue and the correction of the deformity. For scoliosis of the lumbar or thoracolumbar region below T10, the brace is commonly designed using a constant-volume translation principle. The brace shell forms an inclined surface above the iliac crest on the convex side, angling downward and inward. When the patient lies down, the gravitational force of the trunk, combined with the counteracting force of the brace, pushes the convex side of the lumbar spine upwards toward the midline and neutral position, effectively reducing lateral displacement and rotation (126).

#### 3.3.3 Surgical intervention

AIS is a complex three-dimensional spinal deformity. When AIS continues to deteriorate and non-surgical treatment methods fail to achieve the desired therapeutic outcomes, surgical intervention should be considered. We have summarized the current common surgical treatments for AIS, including vertebral fusion surgery, anterior vertebral body tethering (AVBT), growing rod technique, and the SHILLA growth guidance system.

##### 3.3.3.1 Vertebral fusion surgery

Posterior spinal fusion (PSF) is widely recognized as the standard treatment for AIS, particularly for patients with a Cobb angle greater than 40°–50° (127–129). The primary goals of this surgery are to correct spinal deformities, restore balance, and achieve joint fusion, all while minimizing complications (130). Extensive research has demonstrated that PSF can effectively correct spinal deformities and produce favorable outcomes (129, 131, 132). One of the primary costs of surgery is the inevitable restriction of motion at the fused segments. This sacrifice in mobility may increase the risk of degeneration at adjacent segments, but the stability gained from the procedure is crucial for controlling the progression of deformity. At the same time, a key biomechanical benefit of PSF is the complete elimination of the “accordion phenomenon” within the intervertebral discs at the fused levels. This phenomenon is a descriptive term for the dynamic changes of the intervertebral discs in AIS patients under daily, periodic axial loading. The repetitive collapse and re-expansion of the discs, akin to the opening and closing of an accordion, is believed to potentially exacerbate vertebral wedging and the progression of scoliosis through the Hueter-Volkmann mechanism (133). Alongside the development of PSF, anterior spinal fusion (ASF) has emerged. In the 1970s, Allen Dwyer introduced anterior correction techniques for AIS (134). ASF offers benefits such as fewer segments fused and higher fusion rates, but it has limited potential for correcting spinal curvature and may sometimes result in kyphosis (135, 136). Both anterior and posterior spinal fusion surgeries are invasive procedures that can lead to substantial blood loss, extended hospital stays, postoperative pain, and risk of infection (137). To address these issues, minimally invasive spinal fusion techniques have been developed. These techniques minimize trauma and shorten hospitalization times. Researchers such as Sarwahi et al. and de Bodman et al. have successfully implemented minimally invasive spinal fusion for AIS treatment (138, 139). Nevertheless, minimally invasive spinal fusion surgery has limitations: 1. Its corrective potential is limited, requiring careful patient selection based on curve severity (140). 2. There are numerous constraints in performing osteotomies and spinal fusion with minimally invasive techniques (140, 141). 3. Multiple fluoroscopic images are necessary to accurately position rotated vertebrae (142).

##### 3.3.3.2 Anterior vertebral body tethering

Due to the fusion of vertebrae with screws and rods, vertebral fusion surgery may result in restricted growth and movement of the fused spinal segments, as well as degenerative changes and arthritis in the adjacent segments (143–146). AVBT has been developed as a surgical technique. This method is based on the Hueter-Volkmann principle, which states that applying compressive forces to the growth plates slows down skeletal growth, while tensile forces stimulate it, thereby altering vertebral shape (147, 148). In AVBT, tethers and screws are introduced on the convex side of the curved spine to restrict its growth, while allowing the concave side to grow, thus correcting scoliosis. In animal models, Newton et al. found that using anterior-lateral flexible tethers in calf models can regulate spinal growth while maintaining spinal flexibility (149, 150). Multiple studies have shown that AVBT can progressively correct scoliosis in skeletally immature patients with AIS, demonstrating significant therapeutic effects. Additionally, since it does not involve vertebral fusion, it largely preserves joint mobility and muscle strength (151–153). However, AVBT has limitations, being suitable only for skeletally immature patients with mild spinal curvature, and it requires an anterior approach that necessitates opening the thoracic and abdominal cavities, thereby increasing surgical risks (154). Due to its novelty, the indications for AVBT remain controversial, and further research is needed to define precise indications and the specific structures required for patients (155).

##### 3.3.3.3 Growing rod technique

Growing rod technique is indicated for patients with scoliosis who have not responded to conservative treatment, specifically those with a Cobb angle exceeding 50° and aged under 10 years. This technique employs axial traction on the spine, allowing for spinal correction while facilitating continued growth (156–158). However, the requirement for open surgery every six months to extend the growth rod introduces multiple risks associated with anesthesia and surgical incision complications (159–161). The advent of magnetic growth rod technology offers a promising solution to the challenges posed by traditional growth rods, significantly decreasing the frequency of required surgeries. This technology allows for incremental extensions that closely mimic the physiological growth patterns of the spine (162, 163). Magnetic control technology has considerable advantages in clinical applications, as it enables non-invasive manipulation of magnetic devices within the body through magnetic fields to achieve therapeutic objectives. Current research indicates that magnetic growth rod technology can provide adequate traction for scoliotic spines, effectively manage spinal curvature, and reduce both the number of surgical interventions and associated pain, thereby significantly improving patients' quality of life (162, 164, 165). Nonetheless, the weakening and loss of magnetic force present critical limitations for this surgical approach. An increase in body mass index can cause subcutaneous fat to thicken, which increases the distance between the external control device and the magnetic rod. Specifically, for every 1 mm increase in distance, the driving force diminishes by 2.1%, ultimately compromising the traction efficacy of the magnetic rod on the spine (166).

##### 3.3.3.4 The SHILLA growth guidance system

Similar to growing rod technique, the SHILLA growth guidance system can achieve spinal deformity correction while allowing the spine to continue growing. In the SHILLA growth guidance system, all vertebrae are aligned as much as possible in the coronal, sagittal, and axial planes. Then, only the apex vertebrae of the spinal curvature are fused, and non-locking pedicle screws are placed in the remaining vertebrae. These non-locking pedicle screws can slide along the rod, utilizing the growth potential of the patient's vertebrae to guide the vertical growth of the spine while maintaining the normal sequence of the non-fused vertebrae (167, 168). The SHILLA growth guidance system is intended for pediatric patients under the age of 10 who have severe, progressive, and life-threatening scoliosis. It allows for spinal growth while controlling the deformity, eliminating the need for repeated surgical procedures (169–171). However, the sliding of non-locking pedicle screws along the rod can produce metal debris due to wear, which may lead to inflammatory responses (172).

## 4 Discussion

AIS is a three-dimensional spinal deformity located in the coronal, sagittal, and horizontal planes, characterized by vertebral rotation and lateral curvature of the spine, making it the most common type of scoliosis (173–175). In orthopedic-related diseases, biomechanical analysis is an important method that helps us gain a comprehensive understanding of the condition and develop better treatment options (176–178). Therefore, studying AIS from a biomechanical perspective is crucial, as it may assist in developing more effective treatment methods, ultimately improving the quality of life for patients with AIS.

This study systematically reviews the role of biomechanics in the pathogenesis, clinical manifestations, and treatment of AIS, revealing that biomechanical factors are present throughout the entire process of AIS development. These factors serve as the core link connecting abnormalities in the skeletal, muscular, and neural control systems. In terms of pathogenesis, the perspective proposed in this review—that the unique biomechanical challenges posed by fully upright walking represent an evolutionary background for the onset of AIS—aligns with Kouwenhoven et al.'s view that “the uniquely human fully upright posture seems to be a prerequisite for AIS development” (12). However, unlike most reviews that focus on a single factor, such as genetics, molecular pathways, or hormonal imbalances (179, 180), this study innovatively connects molecular biology and biomechanics. It highlights that regulatory molecules such as PKM2 and PAX3 may exert their effects by influencing the biomechanical parameters of the bones (e.g., density, toughness) and the force balance in the spine. This provides a mechanobiological framework for understanding how genetic mutations ultimately translate into macroscopic deformities, bridging the gap between mechanistic studies and clinical manifestations found in traditional reviews. This approach complements the “mechanical load-growth feedback” theory emphasized by Stokes et al (122). In terms of clinical presentation and evaluation, the guidelines of the International Scientific Society on Scoliosis Orthopaedic and Rehabilitation Treatment (SOSORT) and classic literature by Weinstein et al. comprehensively describe the body deformities and functional impairments associated with AIS (64, 93). This review further emphasizes the biomechanical nature of these symptoms, linking abstract symptoms to specific mechanical principles. This perspective strengthens the necessity for biomechanical interventions (e.g., specific exercise rehabilitation), aligning with the core recommendations for exercise therapy in the SOSORT guidelines (93). In the analysis of treatment methods, the conclusions of this study share broad consensus with the North American Spine Society (NASS) guidelines and reviews by Negrini et al., particularly in terms of the core concept of bracing treatment—applying biomechanical forces to alter spinal growth patterns, while surgery serves as the ultimate mechanical reconstruction approach (181, 182). The unique contribution of this study lies in the establishment of an evaluation system based on biomechanics as a unified standard to analyze the mechanical principles and pros and cons of different treatment strategies. For example, with regard to novel non-fusion techniques (such as vertebral tethering), the design concept is entirely based on the mechanical inhibition of the growth plate on the convex side of the deformed vertebra. The biomechanical analysis framework provided by this study offers a solid theoretical foundation for understanding and optimizing such technologies, an area that many traditional treatment strategies have yet to explore in depth (183).

Based on the previous discussions, this review does not repeat the conclusions of existing guidelines. Rather, it organically links the etiology, clinical manifestations, and treatment of AIS through an enhanced biomechanical integrative perspective. It not only supports the core treatment principles based on biomechanics found in existing guidelines, but also introduces biomechanical concepts and systematic mechanical analyses, providing new insights into understanding the complex nature of AIS and offering direction for future development of more targeted biomechanical intervention strategies.
